# Biology of the southern giant hornet, *Vespa soror*: nest architecture, morphological differences among castes, and the genetic structure of colonies

**DOI:** 10.3389/finsc.2023.1136297

**Published:** 2023-07-06

**Authors:** Heather R. Mattila, Lien T.P. Nguyen, Adrien Perrard, Maggie Bain, Gard W. Otis

**Affiliations:** ^1^ Department of Biological Sciences, Wellesley College, Wellesley, MA, United States; ^2^ Institute of Ecology and Biological Resources, Vietnam Academy of Science and Technology, Hanoi, Vietnam; ^3^ Institute of Ecology and Environmental Sciences-Paris (iEES-Paris), Sorbonne Université, CNRS, IRD, INRAE, Universite Paris-Est Creteil (UPEC), Paris, France; ^4^ Sciences du vivant, Université Paris Cité, Paris, France; ^5^ College of Biological Science, University of Guelph, Guelph, ON, Canada; ^6^ School of Environmental Sciences, University of Guelph, Guelph, ON, Canada; ^7^ Institute of Bee Health, University of Bern and Agroscope, Bern, Switzerland

**Keywords:** *Vespa*, giant hornet, tropical hornet, nest construction, colony structure, caste morphology, mating frequency, geometric morphometrics

## Abstract

Giant hornets in the genus *Vespa* are apex predators that are known throughout Asia for their exceptional size and devastating group attacks on social insect colonies. The giant hornets include *Vespa mandarinia*, a well-studied and widespread temperate species, and *Vespa soror*, a poorly known sister species that is limited to subtropical and tropical regions of Southeast Asia. Both species have been recently documented on the west coast of North America, raising urgent questions about their potential impact in novel ecosystems. To better understand the biology of *V. soror*, we describe the nest architecture, caste morphology, and genetic structure of colonies collected in Vietnam. Comparisons of colony metrics between the two giant hornet species suggest important differences that are likely a consequence of the relatively warmer climate in which *V. soror* occurs. Like *V. mandarinia*, *V. soror* constructs large, underground nests of partially enveloped horizontal combs. However, compared to temperate *V. mandarinia* colonies, the longer nesting period of subtropical *V. soror* colonies likely resulted in relatively larger colony sizes and nests by the end of their annual cycle. *Vespa soror* workers and gynes were larger than males, distinguishable based on wing shape and body size (total length and measures of six body parts), and equivalent in size to female castes of *V. mandarinia*. We genotyped colony members from three mature nests, which revealed that males and females were offspring of singly mated queens. Two colonies were monogynous, but one colony was comprised of two unrelated matrilines. Polygyny has not been observed for *V. mandarinia*, but is more common in tropical hornet species. Our study sheds light on essential details about the biology of an understudied species of giant hornet, whose populous colonies and long nesting period suggest the potential for substantial ecological impact wherever they occur.

## 1 Introduction

Hornets in the genus *Vespa* (Hymenoptera: Vespidae: Vespinae) include 22 species of eusocial wasps, most of which are restricted to Asia, with the natural distributions of only two species extending westward to Europe (1, 2). Hornets are impressive predators in their native ranges (3), but some species have gained notoriety after being unintentionally introduced to new habitats, where they have become predators of prey that lack coevolved defenses (4–8). While several alien hornet species have established long-lasting invasions around the world, other introductions have failed, usually for unknown reasons (2, 8–11). Repeated accidental introductions of hornet species into novel habitats have created an urgent need for a deeper understanding of the biology and ecology of all members of this genus to best predict how invasion scenarios might play out (11–13).

While the habits of temperate *Vespa* species are generally well studied, those of tropical species remain relatively poorly known (3, 14–17). Intriguing differences are apparent between hornets inhabiting different biomes. Where they have been documented, nest development follows a similar sequence of phases in temperate and tropical regions, although colony growth is limited by climatic conditions, resulting in generally smaller colonies (numbers of nest cells and individuals) as nesting periods shorten with increasing latitude (16). Hornets in temperate regions typically have an annual cycle that begins when a single queen rears her first generation of workers in a small horizontal comb that she has constructed. After these workers begin to eclose as adults, the colony grows rapidly as they assume the tasks of nest construction, brood care, and foraging. Workers build new combs below the original comb, including larger cells for rearing reproductives, with the number of gynes and males reared related to the size of the colony’s worker population (3, 18). In most species, gynes can be distinguished from workers by their size, but in several species size distributions of female castes overlap (3, 16, 19). In the final phase of nesting, the queen dies and the colony starts to decline. Young gynes leave their nest to mate and, in temperate habitats, they enter a period of winter diapause before founding new nests in the spring. For *Vespa* species in tropical habitats, the timing of nest founding may be asynchronous (15, 16). *Vespa* has traditionally been viewed as having colonies headed by a single queen based on studies of temperate species (14, 16). However, polygyny has been observed frequently in tropical *Vespa* species, and is thought to be driven by heavier predation pressure on incipient nests (14–16).

Available information about the habits of the two species of giant hornets—the northern giant hornet, *Vespa mandarinia* Smith, 1852, and the southern giant hornet, *Vespa soror* du Buysson, 1905—aligns with known differences in the biology of temperate and tropical hornets, although substantially more information is available for *V. mandarinia*. *Vespa mandarinia* is widespread in temperate regions of Asia, occurring from northern Japan to the edge of the subtropics in the highlands of Southeast Asia, and westward to northern India (20–22). Its behavior and ecology have been well characterized based on years of close study in Japan (3, 23 and references therein), where it is an infamous predator of commercially managed honey bees (23, 24). *Vespa mandarinia* individuals have often been described as the largest hornets in the genus. Colonies have a relatively long nesting period compared to other temperate *Vespa* (25), with mature colonies consisting of several hundreds of individuals housed in large, subterranean nests (3, 20, 23). *Vespa mandarinia* colonies are headed by a single queen that usually mates with only one male (26).

In contrast, these biological details have not been well documented for *V. soror*, which is endemic to tropical and subtropical areas of Southeast Asia only, including southern China, Indochina, Thailand, Myanmar, and northeastern India (27, 28). Initially described as a subspecies of *Vespa ducalis* Smith, 1852, then of *V. mandarinia* (19, 20), *V. soror* is now recognized as a distinct species based on morphological characters and a narrow zone of sympatry with *V. mandarinia* (1, 20, 29–31). What is known about *V. soror* comes from anecdotal observations made in Hong Kong (32) and studies of predator-prey interactions between *V. soror* and *Apis cerana* Fabricius, 1793, in Vietnam (33–35). In Hong Kong, Lee’s (32) impression was that *V. soror* workers and gynes were readily distinguishable visually and that nesting habits were similar to *V. mandarinia* (no replicated data were reported). It was speculated that the annual period of development was long based on observations of hornet activity outside nests. Lee (32) was unable to estimate the number of individuals in the mature nest he examined and no one has explored the mating system or genetic structure of *V. soror* colonies. Studies of *V. soror*’s hunting behavior suggest striking similarities with the predation strategy of *V. mandarinia* (33–35). Toward the end of their annual cycles when colonies are rearing reproductives, both species launch damaging group attacks on social insect colonies (23, 32–35).

Recently, *V. mandarinia* has been the focus of intense surveys in the Pacific Northwest of North America following its discovery there in 2019 (10, 36–38). Its potential impact as an invasive species is serious given the vulnerability of commercially managed *Apis mellifera* Linnaeus, 1758, honey bees to attack (23, 24). In Asia, *V. mandarinia* preferentially hunts introduced colonies of *A. mellifera*, which lack the anti-hornet defenses that other native *Apis* species have evolved in response to heavy predation pressure from giant hornets in their shared ranges (8, 24, 39, 40). Also in 2019, a *V. soror* gyne was captured and killed on the west coast of Canada (10). While *V. soror* has not been detected outside of its native range since that time, this single discovery highlights the potential for its transoceanic transport and the need for a better understanding of its biology and ecology (37), which is presently considered lacking (2).

The ability to predict the invasive potential of accidentally introduced hornets is limited by the quality of information that is available about that species in its native range (11–13, 41, 42). Understanding fundamental aspects of a species’ life history is particularly important for giant hornets because group attacks on social insect prey, including economically important *Apis* species, coincide with the onset of the production of hornet reproductives toward the end of their annual cycle (25). Because hornet biology and ecology can differ between temperate and tropical regions (15, 16), well-established details about the northern-ranging *V. mandarinia* may not translate directly to its lesser known southern sister species, *V. soror*. We address this knowledge gap by characterizing *V. soror*’s nest architecture, colony size at maturity, the relative body size and differentiation of workers, gynes, and males, the mating system of queens, and the genetic structure of colonies in Vietnam. Many of these biological traits showcase the close phylogenetic relationship between *V. soror* and *V. mandarinia* and affirm recognition of both species as giants within the genus. Differences that were observed between the two giant hornet species align *V. soror* with other tropical *Vespa* species.

## 2 Materials and methods

### 2.1 Collecting *V. soror* nests and colony members

Three *V. soror* colonies were available to us for study. We purchased Nest #1 (N1) and Nest #2 (N2) on 24 September and 4 November, 2013 from a vespiculturist who had established them in Yen Lap district, Phu Tho Province, Vietnam (21.21 N, 105.16 E). He had excavated these colonies (all combs and colony members) in early June 2013, from nearby subterranean nesting sites, moved them a few kilometers to his home, and installed them within hours of excavation in previously created cavities to minimize colony disturbance. Workers were observed foraging and depositing excavated soil outside of the nest entrances thereafter, which is typical behavior of expanding *V. mandarinia* nests (23). Colonies were left undisturbed until they were re-excavated for us as mature colonies late in their nesting periods. Adult hornets were collected by inserting a PVC tube that had a screened cage at the far end into each nest's entrance. Most hornets flew into the tube and were captured in the cage as they exited the nest. Each nest was subsequently excavated and adults that were still clinging to combs were added to additional cages (see **Supplementary Figure 1** for more details). Thus, we caught most but possibly not all colony members in N1 and N2. Nest #3 (N3) was discovered in an abandoned termite nest in Sop Cop district, Son La Province (20.84 N, 103.31 E). On 12 October, 2020, adult foragers were collected into a screened cage as they returned to their nest. Nest 3 combs were not collected.

After field collections were complete, all hornets (N1–N3) and intact nests (N1 and N2) were returned to the Institute of Ecology and Biological Resources in Hanoi, where the hornets were killed by overnight freezing. The next day, the number of adult individuals collected per nest was counted (N1 and N2) and sorting began for the various analyses described below (colony and sample sizes for each analysis are reported in the Results). Sealed brood cells (pupae) were counted in both N1 and N2 (see Methods section 2.2), although larvae and eggs were not counted. Random subsamples of ~100 workers from each nest (N1–N3) and all reproductive offspring (N2) were pinned for body measures (see Methods section 2.4). Hind legs were removed and preserved in 95% ethanol for DNA extraction (see Methods section 2.5). During this sorting process, we failed to locate the egg-laying queens, although both nests had eggs and larvae so queens, which tend to be large, shiny, hairless, and with worn wings (25, 43), were likely present.

N2 was the only nest in which adult reproductive offspring were present, so individuals were sorted by caste. Males and females were discriminated based on number of antennal flagellomeres and metasomal segments (1). Females were preliminarily categorized as workers and gynes based on size because it has been previously suggested that the two castes could be visually discriminated in both species of giant hornets (23, 32). However, of the ~1,100 females in N2, 14 females were difficult to categorize based on visual impressions of size. We subsequently used a geometric morphometrics approach (see Methods section 2.3), which separates female castes in other *Vespa* species based on overall shape (not size) differences (44, 45), to confirm caste assignments within N2 females. Preliminarily categorized workers and gynes separated distinctly in this analysis and the 14 uncategorized females clustered tightly with other gynes. They were categorized as gynes at this point and included in analyses of caste body sizes thereafter (see Methods section 2.4).

All data generated from the *V. soror* nests (N1 and N2) and hornet specimens (N1–N3) that were examined are available in the **Supplementary Materials** (**Supplementary Data 1**).

### 2.2 Describing the structure of *V. soror* nests

The external envelope was removed from N1 and N2 and the combs from each nest were separated by cutting the connecting petioles. All combs were numbered, from the oldest at the top (comb 1) to the newest at the bottom (comb 5), and then photographed next to a ruler or 10 cm scale to estimate dimensions.

Combs were approximately oval in shape, so their longest and shortest axes were used to estimate comb area. Petioles (also called pedicels or pillars in other published studies) attaching adjacent combs were measured in five dimensions with digital calipers: the widest diameters at both the top and bottom of each petiole, the diameter perpendicular to these measurements, and height (distance between the two attachment points). The top attachment points of the petioles of comb 1 were not recorded for either nest because they widened into the nest envelope and were damaged by its removal. Mean petiole diameter was compared between top and bottom attachment points (paired t-test; SAS proc ttest procedure, version 9.3; SAS Institute, Cary, NC, United States). The relationship between estimated comb area and the number of petioles attaching it to the comb above was also examined across both nests (Spearman’s rank correlation; SAS proc corr procedure).

We counted the numbers of completed cells and sealed pupal cells on each comb in N1 and N2 using digital photographs (Canon G11 Powershot, Canon Inc., Tokyo, Japan) taken the day after nests were collected. These images were also used to estimate cell widths on each comb, which were measured to test Yamane and Makino’s (46) observation that cells tend to be smaller in older parts of *Vespa* nests (i.e., older versus newer comb and central versus peripheral positions). On each comb, we measured cell width for 50 fully constructed cells, including 10 cells approximately in the center of the comb, 10 cells at the periphery, and 30 cells between these two zones. Cells were selected at random from among those that were well positioned for assessment (i.e., cell edges were visible, undamaged, and on a similar plane as the camera). All measurements were made using ImageJ analysis software (National Institutes of Health, Bethesda, MD, United States). Each cell was measured from corner to corner on all three axes (46); these values were averaged to determine mean cell width. For N1 and N2, we determined whether overall cell width (of all 50 cells) differed across combs and whether cell width differed between central versus peripheral zones across combs (one-way and two-way ANOVAs, respectively; SAS proc glm procedure for both analyses). Log transformations were applied to N2 measurements to normalize their distribution; N1 data were normally distributed. We used a Bonferroni correction to lower α = 0.05 because two tests were performed on each dataset; means were separated using Tukey HSD tests. Finally, for both nests we estimated the number of cells (both total and sealed) on each comb that were relatively large (i.e., cells in which gynes could be reared; see body size measures below). These counts were approximate because there was a continuum in cell widths across both nests and we were not able to confirm the caste of larvae and pupae at the time of collection, although many cells were clearly large compared to others.

### 2.3 Assigning females to caste using wing shape

To confirm the caste membership of all females collected from N2 (the only nest in which reproductives were present), we analyzed wing shape using a method of geometric morphometrics that has been shown to separate female castes in other *Vespa* species (44, 46). The right forewing of each specimen was removed at its base and carefully taped flat on a labeled microscope slide. High-resolution photographs of the wings were taken on the same plane as a digital camera mounted on a tripod (Nikon D5100 DSLR, Nikon Corporation, Tokyo, Japan). Slides were photographed on white paper, at night, and with lighting positioned over the slides to standardize image conditions, increase contrast, and eliminate shadows. Each image was imported into tpsUtil software (47) and 19 2D landmarks were placed at the intersections of veins for assessment of the geometric shape of each forewing (**Supplementary Figure 2A**).

We investigated the allometry of the wing venation of workers and gynes from N2 using the R software (48) package ‘geomorph’ (49). A generalized Procrustes analysis (GPA) was used to superimpose the landmarks from all specimens to retain information about their geometric shape only [with location, orientation, and size information having been extracted; (50)]. GPA is an analysis suited to shape data that, unlike a classical ANOVA, is based on Procrustes distances (a metric used to quantify shape differences). Forewing venation size was estimated using the log-transformed (ln) centroid size, a size measurement computed from the 19 landmarks (51). The effects of wing size, specimen caste, and their interaction on wing venation shapes were tested using a Procrustes ANOVA with type I Sums of Squares to test first for size effects, then for caste effects once size effects were taken into account. Procrustes ANOVAs are based on Procrustes distances between specimens’ shapes rather than explained covariance matrices, with a residual randomization permutation procedure for significance testing (52, 53).

To visualize the results, we illustrated the allometric directions of each caste using a scatterplot of the log-transformed centroid size and the common allometric component of the forewing shape, which is a one-dimensional summary of the multivariate shape data using the major axis of covariation between size and shape (54). In such a graph, allometry is indicated by a significant difference from a slope of 0 on a scatterplot of log-transformed centroid size and wing shape. The graph was created in R with ggplot2 (55). The variation of forewing shape across female specimens was also explored using a Principal Components Analysis (PCA).

### 2.4 Comparing body sizes of workers, gynes, and males

Several body measurements were made from workers (N1–N3) and reproductives (N2 only) to examine hornet size and confirm female caste assignments generated by wing morphometrics. For female hornets from all three nests, triplicate measurements of the maximum width of the head and thorax (averaged for each individual) and single measurements of the widths of the first three anterior metasomal tergites were made with digital calipers (nearest 0.01 mm; Marathon Watch Company Ltd., Vaughan, ON, Canada; **Supplementary Figure 2B**). Forewing length was measured for all females and males from N2 (**Supplementary Figure 2A**). Forewings were measured using tpsDig2 for females from N1 and N2 and with calipers for females from N3 and males from N2. For both males and females, body length was measured with calipers from the apex of the head to the apical margin of the second metasomal tergite (**Supplementary Figure 2B**) (56), which is a standard measure of body size for dead wasp specimens that avoids underestimating length due to a curled or contracted gaster. To estimate the total body length of living *V. soror* workers, we captured screenshots from videos of hornets as they attacked *A. cerana* colonies [n = 36 workers captured in 22 videos recorded over two days; videos from (33)]. Body length (head to the end of the gaster, at the tip of the sixth and terminal metasomal tergite) was measured from two images of each hornet when it was landed in a fully extended position near a hive entrance (values averaged per individual; ImageJ; scale set according to the known width of each hive’s entrance).

Estimates of body size were compared among nests and castes in two ways. First, we compared each of the seven estimates of body size among groups (ANOVA and Tukey HSD tests; SAS proc glm procedure). All measures were compared among females; males were included in comparisons of forewing and body lengths only (data were log transformed to improve normality; sample sizes are provided in **Supplementary Table 1**). All measures could be taken from most females, except for a few specimens that were damaged during handling or storage. Six of the body measurements taken from female specimens (forewing length, head, thorax, and metasomal tergite 1–3 widths) were subjected to a PCA that included every individual with a complete set of measurements (N1 = 100 workers, N2 = 82 workers and 40 gynes, N3 = 118 workers). Body length was excluded from this analysis because this measurement was made at a later date and it was not possible at that time to confirm the specimen ID of the gynes. The principal axis method was used to extract the principal components, followed by a varimax (orthogonal) rotation (SAS proc factor procedure) (57). A measurement was considered to load on a given component if the factor loading was greater than 0.40. The first two components were retained and their factor scores were visualized in a score plot.

### 2.5 Mating status of queens and genetic structure of colonies

We explored the mating status of queens and the genetic structure of the colonies for N1–N3 using eight microsatellite loci (VMA-3, VMA-4, VMA-6, VMA-7, VMA-8; LIST2003, LIST2010, LIST2020), which were developed for *V. mandarinia* and *Vespula vulgaris* (Linnaeus, 1758), but are also polymorphic in other vespines (58, 59). Microsatellite profiles were determined for 100 workers from N1; 100 workers, 40 gynes, and 30 males from N2; and 118 workers from N3.

DNA was extracted from the hind leg of each individual (DNeasy blood and tissue extraction kit; Qiagen Inc., Germantown, MD, United States) then amplified in a single multiplexed PCR reaction (BioRad MyCycler, Hercules, CA, United States) (60). The resulting DNA fragments were separated by dye label and size in an automated gene sequencer (Applied BioSystems 3730xl with DS-33 dye set; Waltham, MA, United States) and estimated using open-source, fragment-analysis software (Osiris version 2.14; National Center for Biotechnology Information, National Library of Medicine and National Institutes of Health, Bethesda, MD, United States). All allele calls were made manually to minimize scoring errors that could be generated by automated software routines.

We obtained complete microsatellite allele profiles at all eight loci for every individual that we genotyped, but only seven loci were polymorphic and thus useful for examining the genetic structure of colonies (see Results). At this point, we manually inferred the allele profiles (= genotype) of queen mothers in each nest by identifying the one or two alleles at each locus that were shared by all female (N1 and N3) and male offspring (N2). We assumed all males were derived from queens based on strong evidence that this is the case for *V*. *mandarinia*, *Vespa crabro* Linnaeus, 1758*, Vespa simillima* Smith, 1868, *Vespa analis* Fabricius, 1775, and *V. ducalis* (26, 61–64; see Results section 3.4 for the error probabilities associated with this assumption). A paternal genotype was determined for each female offspring after subtraction of her mother’s alleles. In N3, two groups of females emerged that did not share common alleles at locus VMA-8, which was definitive evidence that there were at least two matrilines in this colony. Queen alleles were inferred within each subfamily of worker offspring, and paternal genotype was determined for each worker thereafter. Within each nest, inferred paternal genotypes were examined for evidence of single or multiple mating by individual queens.

In addition to manual review of the dataset, we checked it for errors using the Microsoft Excel add-in GenAlEx ver. 6.51b2 (65, 66), which was also used to summarize allele number and frequency per locus and generate estimates of observed and expected heterogeneity. For paternity of female workers and gynes, non-detection error (the probability that a male mate went undetected because his genotype was the same as an inferred paternal genotype) and the risk of a non-sampling error (the probability that offspring of a given male were not sampled for genotyping) were assessed for each genetic family (67). For male offspring in N2, we also determined non-detection and non-sampling errors to evaluate our assumption that males were the sons of the queen and not derived from worker-laid eggs (61).

## 3 Results

### 3.1 Architecture of *V. soror* nests

**Figure 1:**
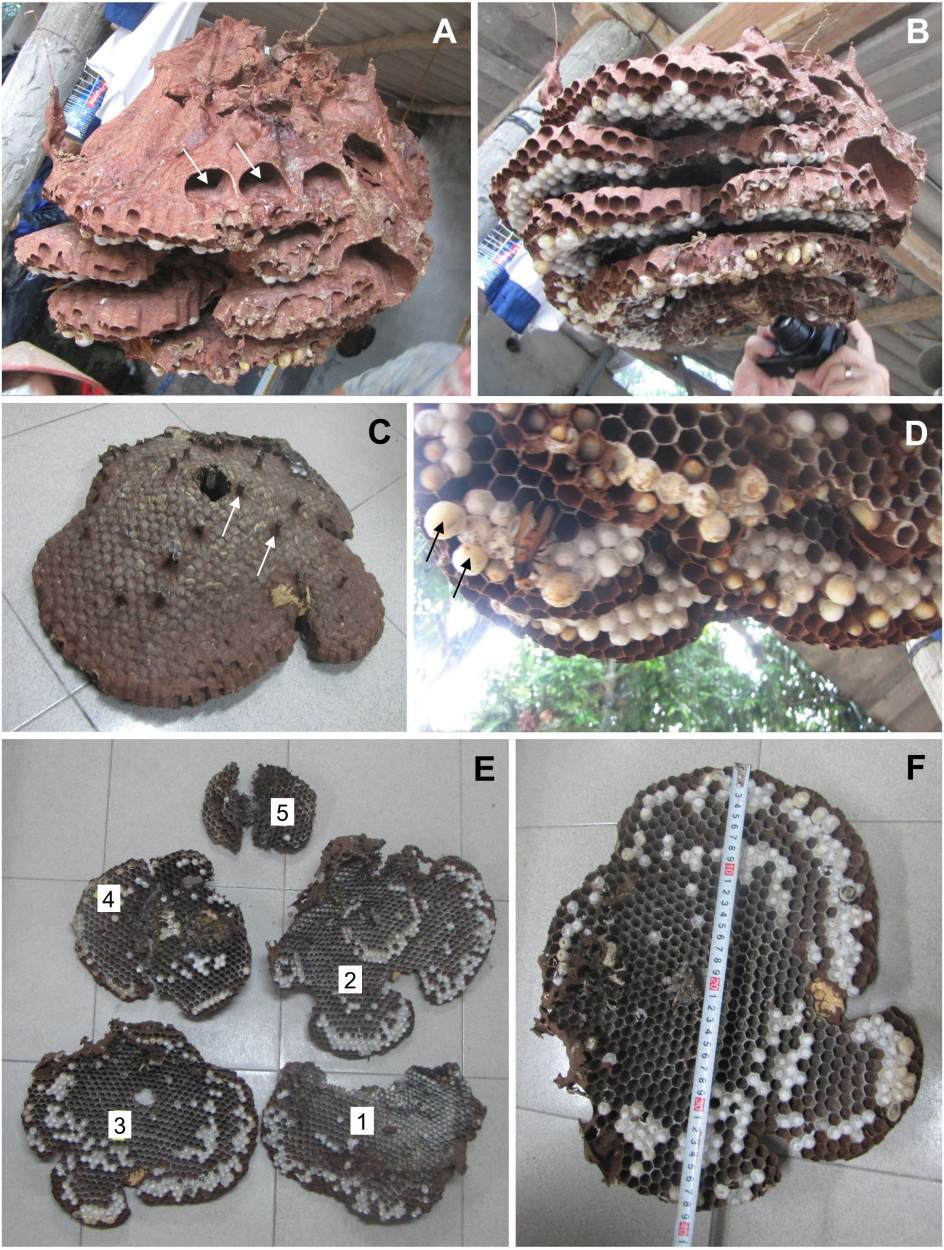
*V. soror* nest N1, collected on 24 September, 2013, in Vietnam. **(A)** The intact nest shortly after removal from the ground, with white arrows indicating the openings of air chambers at the top of the nest envelope and **(B)** again from a lower angle showing cells on the underside of uncovered combs. **(C)** Top view of comb 3, showing the conical shape of the comb and many petioles that connected it to comb 2; two petioles are indicated by white arrows. **(D)** Outer margins of a comb, showing an adult *V. soror* worker and a mix of sealed cells, including a few larger cells that likely contained pupating gynes based on cell diameter and the height of the cappings (black arrows), and smaller cells that contained either workers or males. **(E)** View of the underside of all five combs, numbered from the uppermost, oldest comb (comb 1) to the bottom, newest comb (comb 5). **(F)** Underside of comb 3 showing open and sealed cells, with a measuring tape for scale.

**Figure 2:**
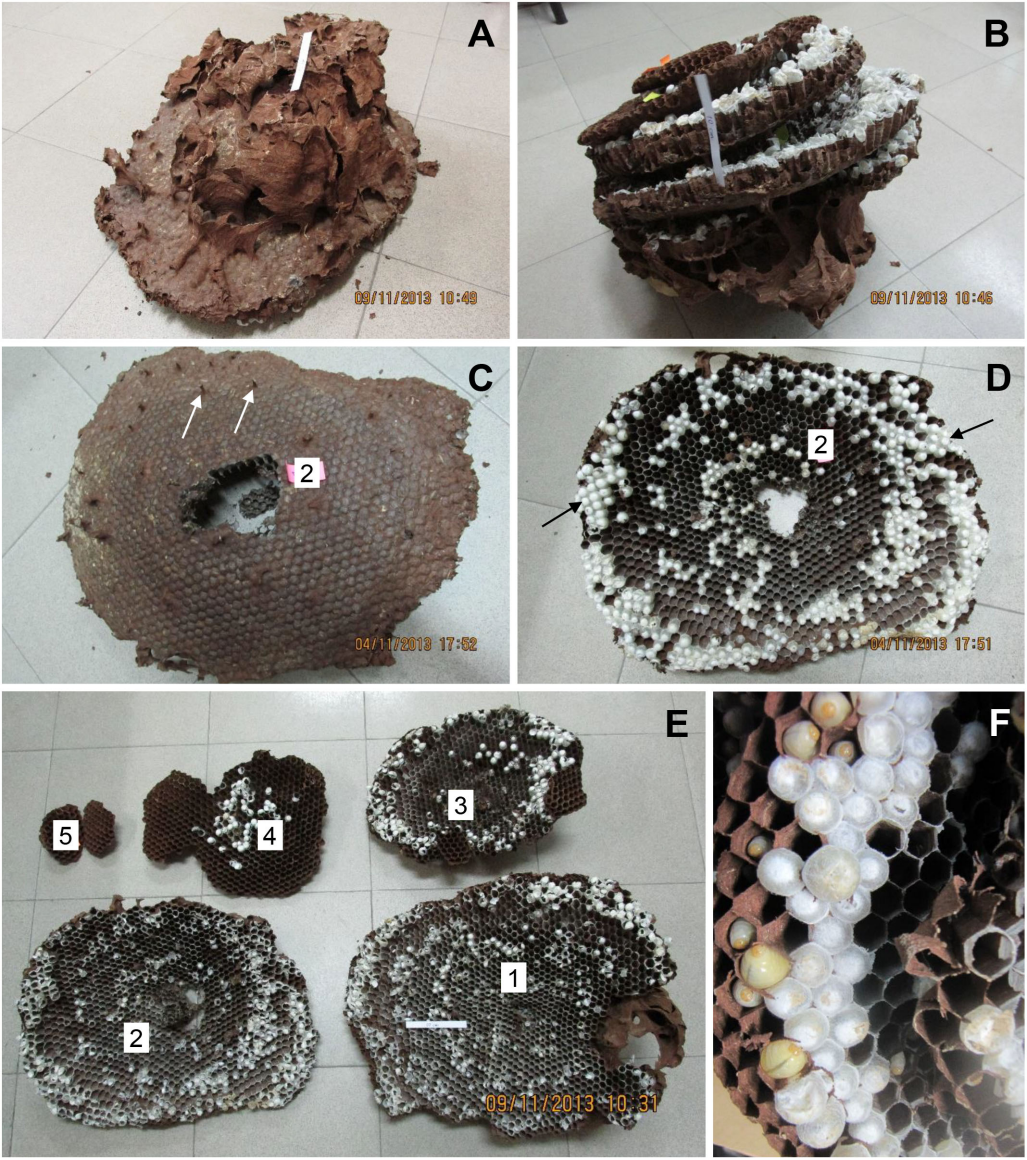
*V. soror* nest N2, collected on 4 November, 2013, in Vietnam. **(A)** The intact nest after removal from the ground, showing the envelope and air chambers from above and **(B)** in an inverted position, showing the stacked combs. **(C)** Comb 2 from above, showing petioles that connected it to comb 1, two of which are indicated by white arrows and **(D)** from below, showing three concentric bands of open cells and sealed brood, smaller (older) central and larger (more recently constructed) peripheral cells, and regions of large sealed cells that likely contained pupating gynes, indicated by black arrows. **(E)** View of all five combs, numbered from the uppermost, oldest comb (comb 1) to the bottom, newest comb (comb 5). **(F)** Underside of comb taken in the field, showing empty cells, cells with larvae, and small and large sealed cells with pupae. A 10 cm strip of paper is included in some images for scale.

Both N1 and N2 had five horizontal combs, with only the uppermost comb 1 (constructed first) and portions of comb 2 covered by an incomplete envelope of thin, papery material (N1: **Figures 1A, B**; N2: **Figures 2A, B**). Above comb 1, both nests expanded into large openings that resembled air chambers (**Figure 1A**). Viewed from the side, each comb was slightly conical in shape, being higher in the center and sloping downward toward the margin, with connecting petioles scattered over the top (**Figures 1C**, **2C**). In N1, combs 1–4 were roughly equal in size and comb 5 was about half their size (**Figure 1E**; **Table 1**). In N2, comb 1 was the largest and the combs below it were successively smaller (**Figure 2E**; **Table 1**). Prior to excavation, we observed workers depositing balls of dirt outside their nest entrances in August, indicating that both colonies were actively enlarging their cavities as nests grew.

**Table 1:** Nest area, number of cells, and number of pupae for N1 and N2.

Comb position	N1			N2		
	Area, cm^2^ (l x w, cm)	# of cells (# large cells)	# of pupae (# large pupae)	Area, cm^2^ (l x w, cm)	# of cells (# large cells)	# of pupae (# large pupae)
1	821 (38.0×27.5)	483 (0)	68 (0)	1858 (55.8×42.4)	1375 (168)	413 (105)
2	1021 (40.0×32.5)	1027 (0)	199 (0)	1371 (48.5×36.0)	1376 (201)	484 (143)
3	1008 (39.5×32.5)	750 (5)	180 (5)	889 (39.7×28.5)	744 (231)	183 (96)
4	915 (37.0×31.5)	706 (0)	144 (0)	720 (33.2×27.6)	554 (0)	41 (0)
5	220 (20.0×14.0)	151 (0)	2 (0)	115 (13.3×11.0)	122 (0)	0 (0)
Total	3985	3117 (5)	593 (5)	4953	4171 (600)	1121 (344)

Comb area was estimated using the formula for the area of an ellipse (area = π ×½length×½width), with length as the maximum comb dimension and width as the maximum dimension at right angles to the axis along which length was measured. Total number of cells and the number of sealed pupal cells were counted on each comb. The subset of pupae and cells that were large (i.e., likely adequate for rearing gynes) was also estimated for both counts.

The dimensions of the numerous petioles that reinforced the stacked structure of adjacent combs in both nests are provided in **Supplementary Table 2**. Bulky and irregular petioles extended from comb 1 to the nest envelope above it. Many petioles attached combs 2–4 to the combs above them, whereas comb 5 was attached by only one or two petioles in both nests, likely because this newest and smallest comb may still have been under construction. We inferred that incomplete petioles were constructed upward because they were not attached to the comb above them. The number of petioles attaching a comb to the one above it was strongly correlated with comb size (Spearman’s rank correlation: *r*_s_ = 0.81, P = 0.015; only completed petioles were counted for n = 8 combs across N1 and N2; **Supplementary Figure 3**). Petioles ranged from nearly circular to strongly oval in cross-section and were larger on average at the base than at the top (mean diameter 9.2 ± 5.1 mm versus 7.7 ± 3.8 mm; paired t-test: t = 4.0, df = 96, P < 0.0001).

N1, collected in late September, had an estimated comb area of almost 4,000 cm^2^ and just over 3,000 cells, 19% of which contained pupae (**Table 1**). At this stage in its development, only 1% of the sealed cells were large enough to hold larger pupating gynes (**Figures 1D, F**), suggesting that no adult gynes had yet emerged. The vast majority of the sealed cells (99%) were smaller and likely contained workers and possibly males based on body sizes (see below), although no adult males were present in this colony. Unsealed cells comprised a mix of empty cells and cells that contained eggs and larvae (**Figures 1D, F**). One day after N1 was collected, large larvae were observed scraping the sides of their cells with their mandibles, a food-begging behavior known from other *Vespa* species, which appeared to be synchronized among neighbors (**Supplementary Video 1**) (68, 69). N2, collected in the same location in early November, was 20% larger than N1, with almost 5,000 cm^2^ of comb and more than 4,000 cells, 600 of which on the periphery of the lower three combs were large enough to contain gyne pupae (**Figures 2D, F**; **Table 1**). At this later point in its development, N2 had almost twice as many sealed pupal cells as N1, and 31% of these cells were large enough to contain developing gynes. Pupae in smaller cells were probably a mix of workers and males, both of which were present as adults. Many larvae were still present in N2 at this late collection date (**Figure 2F**). Collected six weeks earlier, N1 had only 40% of the adult population size of N2 (N1 = ~480 colony members versus N2 = ~1,200 colony members, including 85 males and 53 gynes) and about half the number of sealed cells (**Table 1**).

**Figure 3:**
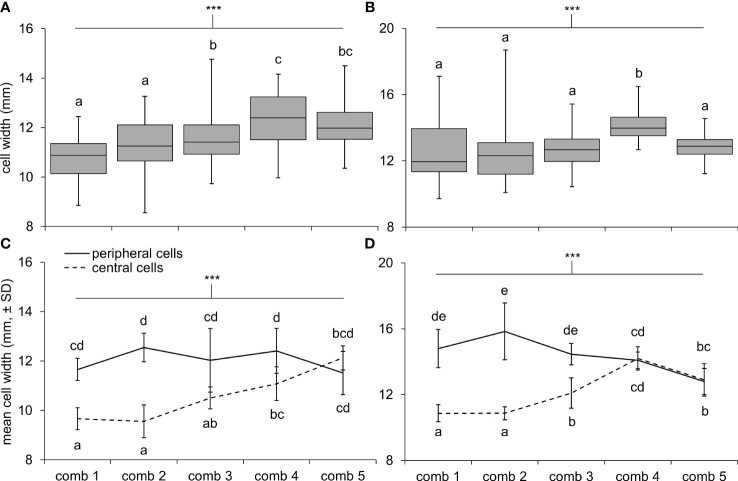
Cell widths differed across comb locations in N1 and N2. Box and whisker plots show the range of cell widths for 50 cells measured across each comb of **(A)** N1 and **(B)** N2: 10 cells in the center, 10 cells on the periphery, and 30 cells in the area between these zones. Differences in the mean widths of cells at the center and periphery of combs were greater in older combs compared to newer combs (i.e., comb 1 is the oldest comb, comb 5 is the newest comb) in **(C)** N1 and **(D)** N2. Asterisks indicate a highly significant effect of comb number **(A, B)** and interaction effect between comb number and zone **(C, D)** with a Bonferroni adjustment to the level of significance after conducting four ANOVAs (α = 0.0125; P < 0.0001 in all cases); letters indicate differences between means within each panel, according to Tukey HSD tests.

Cells were hexagonal but irregularly so, with widths on three axes often differing 1–2 mm per cell. In general, cell widths (averaged across the three axes) also varied tremendously within nests (**Figure 3**). In both nests, newer combs had larger cells on average than older combs (**Figures 3A, B**; one-way ANOVAs; N1: F_4,245 _= 17.3, < 0.0001; N2: F_4,245_ = 11.5, P < 0.0001). Also, cell widths tended to be larger at the periphery of combs compared to the center (**Figures 3C, D**; two-way ANOVAs, zone effect; N1: F_1,90 _= 97.5, P < 0.0001; N2: F_1,90_ = 170.5, P < 0.0001). However, the extent of this difference depended on the age of the comb, with cell widths between zones becoming homogenous on more recently constructed combs (comb and interaction effects; N1: F_4,90 _= 8.8, P < 0.0001 and F_4,90_ = 16.3, P < 0.0001; N2: F_4,90_ = 8.7, P < 0.0001 and F_4,90 _= 39.7, P < 0.0001).

### 3.2 Assigning females to caste based on wing shape

**Figure 4:**
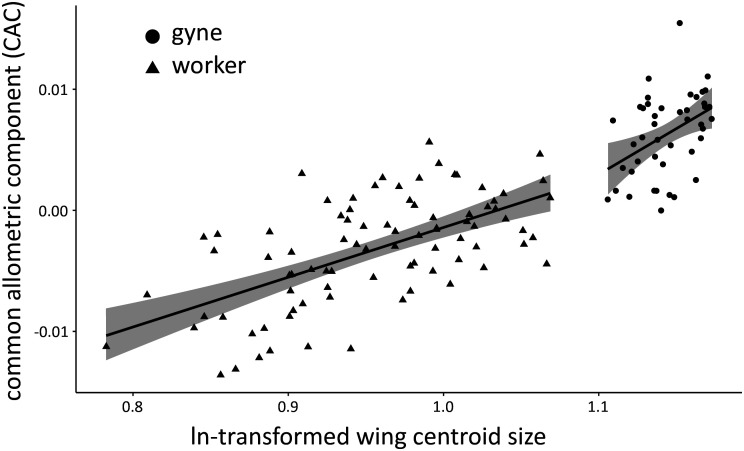
Multivariate regression of overall wing-shape allometry based on 19 wing venation landmarks measured for the forewings of workers and gynes from N2 (n = 86 and 45 specimens, respectively). The graph shows the regression lines (black) of both groups with their 95% confidence interval (gray). Global wing-shape allometry was significant (P = 0.001), as were caste-related differences in wing shape (P = 0.011). The interaction of size and caste was not significant (P = 0.64).

We examined the wing shape of females to confirm caste membership with geomorphic morphometrics. The centroid sizes of wings showed a clear separation of N2 females into two groups (workers and gynes), with a break between clusters at ln wing centroid size = 1.09 (**Figure 4**). These castes assignments were affirmed by comparisons of other body measurements between these two groups (see next section). Wing shape allometry was significant, but there was also a significant caste-related wing shape difference once shape variation related to allometry was taken into account (**Table 2**). Even though the allometric slopes appeared to be graphically different between the two castes (**Figure 4**), the interaction between size and caste was not significant in the analysis (**Table 2**).

**Table 2:** Results of the Procrustes ANOVA testing the effects of size and caste on wing shapes quantified by geometric morphometrics.

	df	SS	MS	Rsq	F-value	Z	P-value
ln(Csize)	1	0.00332	0.00332	0.06425	8.77	7.16	0.001 *
caste	1	0.00070	0.00070	0.01358	1.85	2.26	0.011 *
ln(Csize) × caste	1	0.00033	0.00033	0.00641	0.87	-0.31	0.64
residuals	125	0.04730	0.00038	0.91576			
total	128	0.04166					

Specimen size was estimated by the log-transformed (ln) centroid sizes (Csize) of its wing. The interaction effect of the two independent variables was also included. Asterisks indicate a significant effect.

A PCA of the landmark dataset for wing shape showed caste differences in the two first components of variation for females from Nest 2 (**Supplementary Figure 4**). Most wing shapes for gynes were in the positive values of these two components, while most worker shapes were in the negative values of at least one of these two components. The two first principal components explained 21% of the total shape variation (11% and 10%, respectively).

### 3.3 Body size of workers, gynes, and males

**Figure 5:**
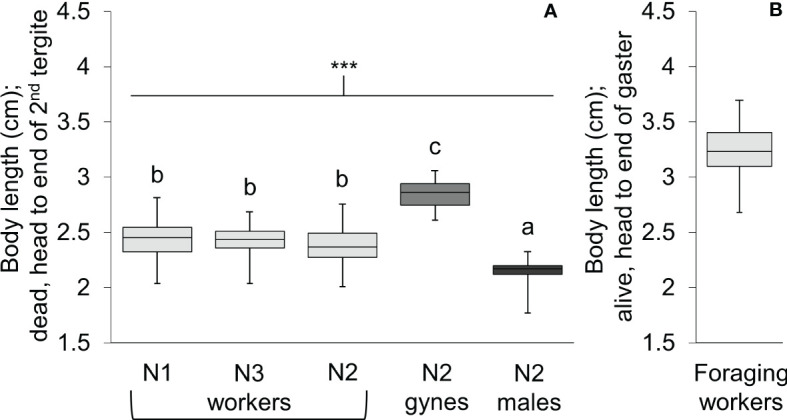
Box and whisker plots of body length for *V. soror* workers, gynes, and males. **(A)** Body length of individuals from the three castes were compared across N1–N3 using a standard measure for dead wasps (head to the apical margin of the second metasomal tergite). Asterisks indicate a highly significant difference across groups (P < 0.0001); letters indicate differences between means according to a Tukey HSD test. **(B)** Total body length (head to the end of the gaster, the tip of the sixth metasomal tergite) of foraging workers was determined from videos of them landed on the front of *Apis cerana* hives (based on known dimensions of hive entrances).

**Figure 6:**
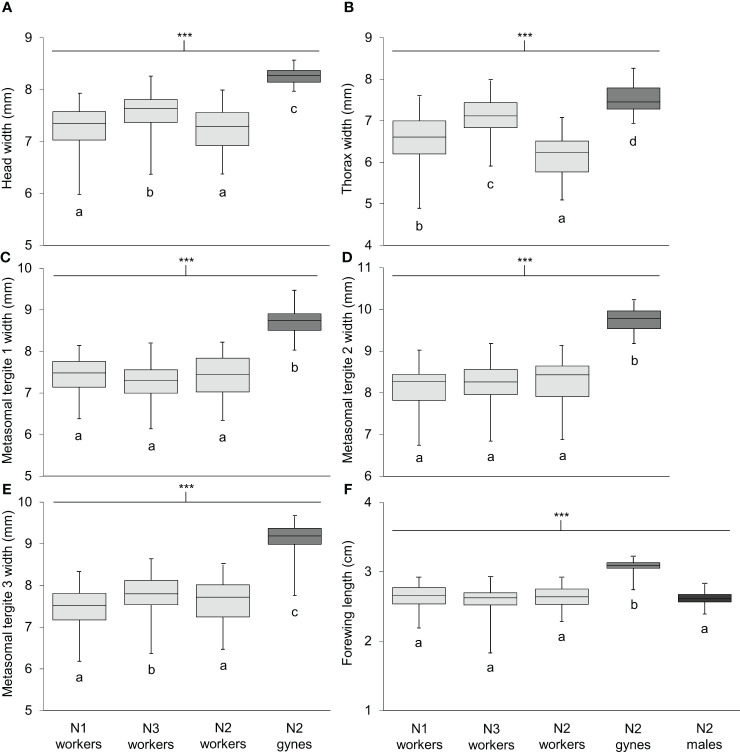
Size of six body parts measured on specimens of *V. soror* workers and gynes, and forewings of males. Characters compared included **(A)** head width, **(B)** thorax width, **(C–E)** widths of metasomal tergites 1–3 and **(F)** forewing length. Workers were collected from N1–N3; gynes and males were present in N2 only. Sample sizes and outcomes of one-way ANOVAs are provided in **Supplementary Table 1**. Body measurements are shown in **Supplementary Figure 2**. Asterisks indicate a highly significant effect of caste/sex on size in all cases (P < 0.0001); letters indicate differences between means within each panel according to Tukey HSD tests.

Body length differed significantly among workers, gynes, and males (**Figure 5**; see **Supplementary Table 1** for ANOVA outcomes). Gynes were larger than workers and workers were larger than males. Workers had similar body lengths across the three nests. In general, these trends held when the mean sizes of body parts were compared across castes from different nests (**Figure 6**; **Supplementary Table 1**). These size differences support our assumption that large pupal cells with strongly raised cappings at comb peripheries were likely gynes and that males are reared in smaller cells. For each character measured, there was some overlap in size between the largest workers and the smallest gynes; the least overlap was detected in the width of the second metasomal tergite (**Figure 6D**).

**Figure 7:**
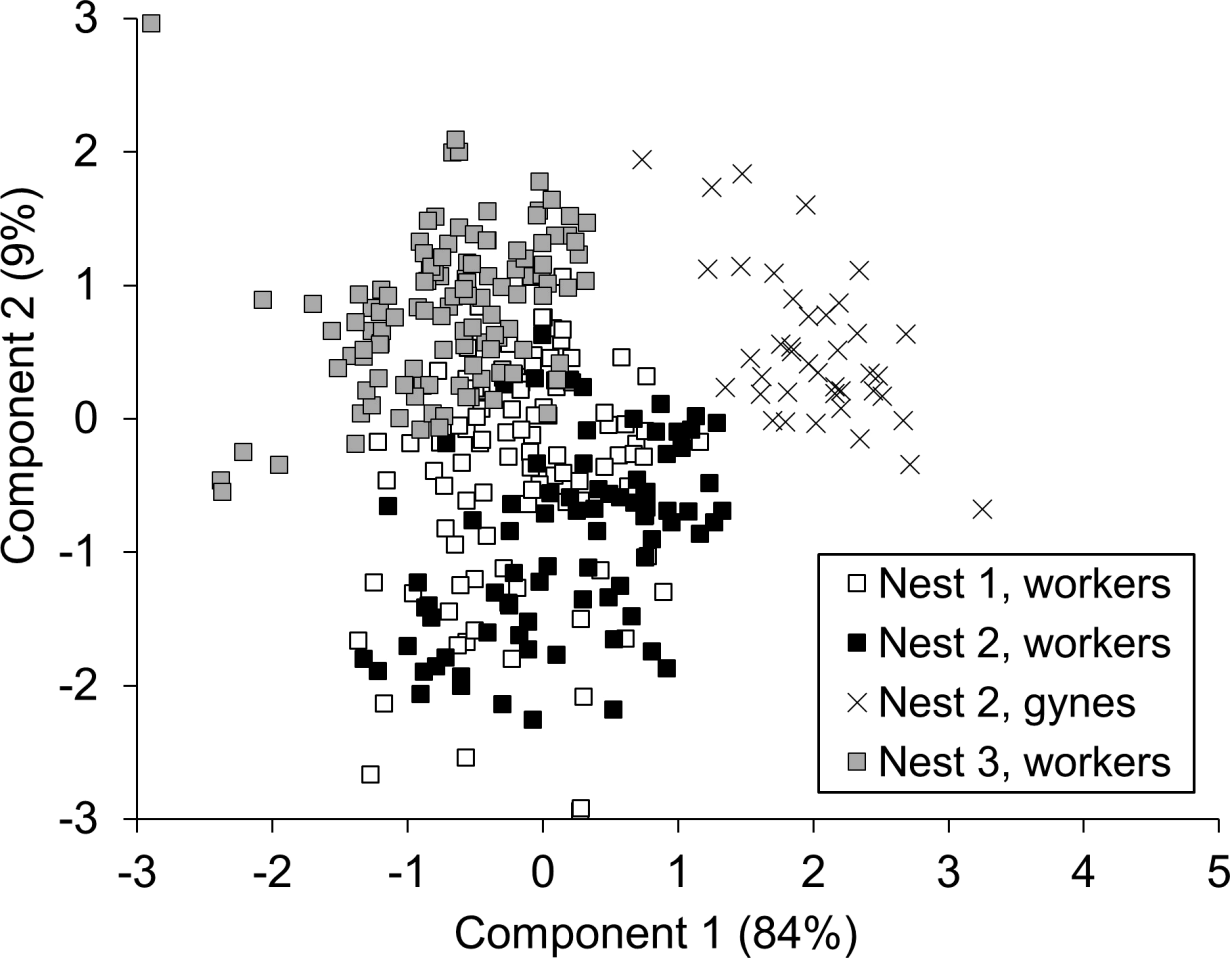
Score plot for the first and second components of a PCA of body size for *V. soror* females. Only individuals for which all six body parts (head, thorax, and metasomal tergite 1–3 widths, plus forewing length) had been measured were included in the analysis; n = 100, 82, and 118 workers were included from N1–N3 and n = 40 gynes from N2.

A PCA was conducted on six body measures taken from females. The first principal component accounted for 84% of the total variance and loaded on five of the six measurements (all but the width of metasomal tergite 2). The second principal component accounted for 9% of the total variance and loaded primarily on metasomal tergite 2. Together, the first two components accounted for 93% of the total variance. When factor scores for these components were plotted against each other, workers and gynes clearly separated from each other and workers from different colonies partially overlapped (**Figure 7**). This pattern confirmed the outcome of female caste identification via wing morphometrics (**Figure 4**).

### 3.4 Mating status of queens and genetic structure of colonies

Seven of the eight microsatellites had moderate to high levels of polymorphism (**Table 3**), with mean 4.6 alleles per locus and H_e_ of 0.63, supporting a decisive assessment of the genetic structure of colonies. One locus (VMA-4) was monomorphic across all specimens, rendering it uninformative for genotyping. A small number of workers were identified in N1 (3 workers) and N3 (2 workers) that had drifted from other colonies, so identified because each worker had unique alleles at a minimum of four microsatellite loci that were not shared by any other individual in their nest (including other drifters). These drifter genotypes revealed allelic diversity beyond that detected among the genetic members of N1–N3 (**Table 3**).

**Table 3:** Number of alleles, allele frequencies, and expected and observed heterozygosity (H_e_ and H_o_) for each of the loci used to genotype colony female offspring from N1–N3 (353 females were successfully genotyped: N1 = 97 workers; N2 = 140 workers + gynes; N3 = 116 workers).

Locus	n	Allele frequencies	H_e_	H_o_
VMA-3	4 (6)	0.399, 0.340, 0.163, 0.098	0.69	0.84
VMA-4	1 (1)	1.00	0	0
VMA-6	2 (3)	0.579, 0.421	0.49	0.56
VMA-7	4 (6)	0.542, 0.156, 0.153, 0.149	0.64	0.78
VMA-8	9 (10)	0.252, 0.222, 0.137, 0.109, 0.089, 0.072, 0.067, 0.026, 0.026	0.84	1.0
LIST2003	3 (4)	0.541, 0.433, 0.026	0.52	0.89
LIST2010	6 (7)	0.433, 0.232, 0.211, 0.098, 0.013, 0.013	0.70	0.64
LIST2020	4 (4)	0.646, 0.205, 0.082, 0.067	0.53	0.58

Summary values were determined by pooling samples across nests into a single population to provide general information for *V. soror* about the allelic diversity of these microsatellites. Allele number (n) and frequencies were determined for colony members only (drifters were excluded); total allele numbers when five drifters were included were also provided in parentheses to indicate potential diversity beyond these three nests.

Genotyping indicated that all sampled offspring in N1 and N2 were produced in each nest by a single queen that had mated with only one male (**Table 4**). No more than one potential queen and male mate was necessary to account for offspring genotypes. In contrast, N3 had two queens, which was evident from differences between matrilines in inferred alleles for five of seven loci. Critically, N3 matrilines did not share any alleles at VMA-8, confirming that the queens were unrelated. Like N1 and N2, both N3 queens had mated with a single male (**Table 4**). One queen dominated production of sampled offspring in N3 (**Table 4**).

**Table 4:** Nest-level summary of queen number and mating status for N1–N3.

Nest	Queen #	# of male mates per queen	Proportional contribution per queen	% non-detection error per queen
N1	1	1	1	0.9%
N2	1	1	1	1.5%
N3	1	1	0.85	0.6%
N3	2	1	0.15	0.5%

All queens were singly mated, with a very low probability of non-detection of additional male mates within each matriline. N1 and N2 had one queen each and N3 had two queens; the proportional contribution of each queen to offspring production is given per nest.

Because we examined a robust number of microsatellites and sample sizes for females were large, the probability that a male mate went undetected within a matriline (a non-detection error) was very low (≤1.5%; **Table 4**) and the probability that a second male’s offspring were not sampled (a non-sampling error) approached zero (67). There was no evidence of worker-derived males in N2. Four of the loci had “informative alleles”, meaning the paternal alleles that workers inherited from their father were not shared by the queen (61). Based on this number, the possibility of assigning a male to the queen when in fact he was derived from a worker was 6.3% (non-detection error). This potential error translated into variable probabilities for missing worker-derived males among the 30 males we genotyped (non-sampling error). For instance, if workers produced 2% of males, the probability that these males were not sampled would be 57% (**Supplementary Figure 5**). However, if workers produced 10% of males, this probability dropped to 5%.

## 4 Discussion

*Vespa soror* is an impressive hornet species that is worthy of the designation as a giant within the genus. Everything about it is big: members of both female castes and males are strikingly large, their colonies are populous, and mature nests are expansive. Despite being relatively common within its range, little has been published about the biology of this conspicuous species. Our study of a small number of *V. soror* nests and hornet specimens from Vietnam showcases strong biological similarities to the other giant hornet, *V. mandarinia*. As a starting point, workers of the two species are equally large [(70); and see below]. Beyond the goliath size of the hornets themselves, our study suggests that *V. soror* nest size (number of cells) and colony size (number of individuals) meets or exceeds that described for *V. mandarinia*. Large nests and colonies sizes may be permitted by the longer nesting period in *V. soror*’s relatively warmer subtropical habitat. In the same vein, our discovery that *V. soror* colonies can be polygynous contrasts with the mating system reported for *V. mandarinia*, but is aligned with reports of polygyny in many tropical *Vespa* species. We elaborate on this comparative biology below and call for continued study of more *V. soror* specimens to confirm these patterns.

Few details have been published about the colony cycle for *V. soror*. However, the status of the mature nests we described and observations by Lee (32) of nest entrance activity allow a comparison with *V. mandarinia* of inferred annual milestones. In southwestern Japan, *V. mandarinia* gynes start nesting in May or June, colonies begin to rear reproductives in August, queens disappear by October at the latest, and colony activity ceases in November (23, 25). Although records from warmer parts of *V. mandarinia*’s range are scant, giant hornet farmers in subtropical regions of India first observe foraging queens in late April or early May and they harvest mature nests 4–6 months later (71). In comparison for *V. soror*, by late September in the subtropical climate of northern Vietnam, workers in N1 had only recently started rearing reproductives. N1 had eggs and larvae of all ages, only five pupal gynes, and all adults were workers, suggesting the queen was present and the colony was just entering its reproductive period (25). Collected six weeks later in early November, N2 was in the midst of its reproductive period, with over a thousand pupae, including hundreds of pupal gynes, and a modest number of adult reproductives of both sexes. There were still large numbers of larvae present as well as some eggs. The presence of many workers in this mature colony suggests that care for larval reproductives was ongoing and the colony had not reached the end of its active season, which would conclude with the disappearance of the queen, a halt to brood rearing, and departure of remaining adult reproductives (25). Differences between the composition of the two nests in Vietnam align with Lee’s (32) impression in Hong Kong of rapid colony growth of *V. soror* colonies from September through November, with activity ceasing in December or January. Collectively, these observations suggest that *V. soror*’s nesting period is longer in the subtropics than that of temperate-nesting *V. mandarinia*.

*Vespa soror*’s longer nesting period likely enables the remarkably large colony sizes we observed. N1, collected in late September, had about 500 adults (workers only), whereas N2 contained around 1,200 adults (workers plus reproductives) in early November, and many more individuals were still being reared (including over 1,100 pupae). In comparison, mature *V. mandarinia* colonies in temperate climates are reported to be much less populous, producing fewer than 1,000 individuals throughout the entire cycle of a colony (46). In central Japan, worker numbers typically peak in September at a few hundred adults [e.g., mean 179, maximum 514 workers (25); maximum 540 workers (72)] and in November for reproductive adults [e.g., mean 74 and maximum 396 gynes; mean 74 and maximum 284 males (25)]. *Vespa mandarinia* colonies in northern Japan had no more than a couple hundred individuals at maturity (46), which is approximately the size of the largest invasive *V. mandarinia* colonies captured in the United States (70). Lee (32) did not census the number of individuals in *V. soror* colonies in Hong Kong, but his impression was that colonies were large based on entrance activity. Our specimens provide the only known estimates of the size of mature colonies for this species. These limited data suggest that *V. soror* nests are impressively large in size at peak maturity compared to *V. mandarinia* colonies surveyed in temperate climates. Colonies of both giant hornet species may be larger in warmer climates, which is observed in other *Vespa* species (15, 73, 74). For instance, mature *V. mandarinia* colonies in subtropical India are reported to have over 1,000 individuals and 8–9 combs that are similar in size to the ones we documented here (**Table 1**) (71). Nests on the island of Taiwan are described as having up to 10 combs (73). It has been noted that subtropical and tropical vespines produce larger colonies in comparison to their temperate counterparts, attributed mostly to abundant food resources fueling greater colony growth over a longer nesting period (3). Colonies could also grow larger if rate of brood development is faster in warmer zones because of a higher rate of prey intake, or higher soil and brood-nest temperatures. Temperature is less likely to drive more rapid colony growth because *Vespa* species appear to be able to maintain consistent brood-nest temperatures of ~30°C across conditions (3, 75, 76).

Potential differences in mature nest size (number of cells) were not as strongly evident between the two giant hornet species, although the *V. soror* nests we examined were on the large end of what has been observed for *V. mandarinia* nests. However, the excavation of more *V. soror* nests is needed to confirm this impression because the structure of only three mature nests has been described so far, which is exceedingly few compared to *V. mandarinia*. For example, Matsuura and Koike (72) report assessing 1,756 *V. mandarinia* nest sites over five decades, providing one of the best examples of the knowledge gap between the two giant hornet species. The two *V. soror* nests that we examined both had five combs with 3,117 and 4,171 cells in total. Lee (32) excavated a third mature *V. soror* nest and counted roughly 2,700 cells, also across five combs. These sizes fall within the upper range of what has been reported for *V. mandarinia*. For instance, nine *V. mandarinia* colonies collected at the end of the active season in southwestern Japan ranged in size from 1,326–4,661 cells (23). A larger sample of 15 colonies from the same region, made in October, yielded a mean nest size of 2,712 (± 985 SD) cells (25). Two mature nests from northern Japan had only 675 and 1,141 cells (46), and four mature nests collected at a similar latitude on the west coast of North America ranged from 418–1,329 cells each (70), suggesting a possible increasing size gradient for giant hornet nests as habitats warm toward the equator (30). Across accounts, mature *V. mandarinia* nests consist of four to ten combs (23, 25, 46, 70, 71), which is in line with the comb number observed for the *V. soror* nests described to date. Vespine nests are known to vary considerably for the same species within the same region and depending on nest site (3), and cells are reused for larval rearing (46), so more information is necessary to determine whether the size of the nest itself (cell number) can reflect the potential for colony size (number of individuals) as much as the duration of the annual cycle does. Furthermore, it is possible that polygyny in *V. soror* colonies (discussed below) could be associated with the production of populous colonies because higher levels of intracolony genetic diversity are linked with larger colony sizes within the vespines (77).

Other traits of *V. soror* nests align with what is observed in *Vespa* generally and giant hornets specifically. *Vespa mandarinia* nests are most commonly found in forests and near “green spaces” (25, 46, 78, 79), which is similar to the type of habitat where *V. soror* nests were discovered in Hong Kong (32) and Vietnam (by LTP Nguyen). The vast majority of *V. mandarinia* nests are subterranean (3, 22, 25), with rare reports above ground (20, 46, 70, 72), and present indications are that *V. soror* has a subterranean nesting preference as well. Lee (32) observed a few colonies that appeared to be living aboveground in human-built structures, but he could not confirm nest presence. Subterranean nests offer relatively consistent conditions and allow for incomplete envelopes, the condition described for nests of both giant hornet species and other subterranean vespine nests (46). As with *V. mandarinia*, *V. analis*, and *Vespa tropica* (Linnaeus, 1758), combs of *V. soror* nests were “umbrella like” or conical in shape, with an uneven upper surface, a large petiole that connected combs centrally (sometimes called the mainstay), and numerous auxiliary petioles for additional support (3, 25, 46). Finally, cell widths in *V. soror* nests reported here (9.7–15.8 mm) were similar to the ranges reported for *V. mandarinia* [9.1–15.5 mm (23); 10.1–15.2 mm (46)]. As in other vespine genera [e.g., *Vespa* and *Vespula* species (25, 46, 80, 81)], cell sizes in *V. soror* nests increased as combs were constructed downward and outward; the smallest cells were the oldest, located in the center of the uppermost comb built by founding queens, and subsequent worker-built cells became progressively larger over the season. The construction by *V. soror* workers of larger gyne cells at the comb periphery is also typical of the nests of *V. mandarinia* and other vespines (46, 80).

Individuals of both giant hornet species are equally large. *V. soror* workers were mean 3.2 cm long from their head to the tip of the abdomen (range 2.7–3.7 cm; **Figure 5B**), which is comparable to similarly measured *V. mandarinia* workers [data from two *V. mandarinia* nests: mean 3.0 cm, range 2.2–3.7 cm, n = 138 workers (70)]. Any size differences between these two species may be an effect of climatic differences. An increase in worker size or colony size with increasing distance from the equator (e.g., Bergmann’s rule *senso lato*) could be hypothesized, but it has proved inconsistent within the social insects (82–86) and insects more broadly (87), and would require a greater sampling effort to be tested on these two hornet species.

Female castes of *V. soror* were clearly discriminated by geometric morphometric analysis of wing shape. Moreover, gynes were 16% larger on average than workers (head to apical margin of second metasomal sternite). Previous authors have stated that female castes of most *Vespa* species are often reared in cells that are distinctly bimodal in size and can be discriminated visually as adults (16, 17, 19, 88), including species of giant hornet (22, 31) [exceptions: *V. tropica* and *V. analis* in Malaysia (89)]. We generally support this assertion for *V. soror* based either on the size of body parts or wing-shape variation. The latter result suggested that gynes are not simply larger workers, but that developmental differences exist among female castes (44). Nonetheless, it may be challenging to visually discriminate between workers and gynes in the field. We found it difficult to do so for a handful of female specimens from N2 until we applied quantitative techniques to body measurements. Body weight, which we did not measure, would be another helpful diagnostic trait (90–93); it reliably separated castes in four *V. mandarinia* nests that had intermediately sized females [(70); see also (23)].

The genetic structure of colonies revealed an intriguing alignment of *V. soror* with the mating frequency of temperate *V. mandarinia*, but simultaneous divergence toward polygyny known from tropical *Vespa* species. Across all three colonies, each *V. soror* queen had mated with a single male and there was no evidence of worker-produced males, which agrees with other reports of *Vespa* queen monandry, worker policing, and near absent ovarian development among workers in queenright colonies [e.g., *Vespa affinis* Linnaeus, 1764; *V. analis*; *V. crabro*; *V. ducalis*; *V. simillima* (11, 26, 43, 61–64, 94–96)]. However, one *V. soror* colony was polygynous, with worker matrilines derived from two unrelated queens. Monogyny and haplometrosis have long been considered the rule among vespines, although reports of polygyny via pleometrosis (founding of a nest by two or more functional queens) and secondary polygyny (adding queens after nest founding) have accrued over time for *Vespa* and *Vespula* (14, 16, 97). These “exceptions” occur mostly in the tropical or warmer parts of a species’ range [e.g., *V. affinis* and *V. tropica* in Sumatra and New Guinea (14, 15, 98)], where coexisting functional queens may facilitate better protection against predation and faster growth than is possible in monogynous colonies (14, 99). The infrequent detection of double matrilines in temperate *V. crabro* colonies has been attributed to spring nest usurpation (61), when a founding queen is displaced by another queen as she establishes her first generation of workers (16, 100). Usurpation was invoked to also explain the occurrence of multiple matrilines in *V. analis* and *V. ducalis* colonies collected in September in Japan (without confirming the presence of multiple queens) (62, 96), although the first queen’s workers would not be expected to persist past the rearing of the second queen’s reproductives (15, 97). We cannot rule out usurpation, but it is more likely that two queens coexisted in the multiple-matriline *V. soror* colony (N3), given that it was sampled relatively late in its nesting period. With one of the three nests we studied containing multiple matrilines, more sampling is warranted to accurately estimate the frequency of polygyny in *V. soror*. Of interest, people in southern China place 2–4 overwintered *V. mandarinia* queens together in a large box in the spring to obtain extremely large multi-queen colonies that they harvest in the fall (S Dong, pers comm), indicating that giant hornet queens can tolerate each other for an entire annual cycle.

We have detailed some basic biological features of *V. soror* colonies inferred from dissections of their nests and analyses of adult hornets in Vietnam. Because young *Vespa* queens typically spend several months in diapause during winter (3), they can easily be transported to distant localities (11) where they occasionally establish invasive populations [e.g., *V. velutina* in Europe (7); *V. tropica* in Guam (101)]. The biology of *V. soror* suggests that its invasive potential is similar to other hornet species, with the risk of establishment potentially higher if transported colonies were to bring with them more genetic diversity due to polygyny (11). If successfully established, populous and long-lived *V. soror* colonies would impose substantial predation pressure on local prey species. The collection of a single *V. soror* queen in 2019 in Vancouver, Canada, confirms that this giant hornet species has the potential to be accidentally introduced to exotic locales (10, 11, 37). In recent years, the discovery of whole nests of *V. mandarinia* in western North America has highlighted the potential for human-mediated dispersal of giant hornets from Asia (10, 11, 37, 38), raising deep concerns about risks faced by beneficial insects and humans. Both giant hornet species occur naturally in forested habitats that can include urban green spaces (23, 32, 72, 78, 79), and modeling suggests that suitable habitat for *V. mandarinia* occurs on all continents except Antarctica (41). Niche modelling has not been conducted for *V. soror* as it has been for *V. mandarinia* (13, 41, 42, 102), although the more subtropical distribution of *V. soror* suggests that it would require relatively warmer regions. The capacity of *V. soror* to destroy economically important social insect colonies (e.g., honey bees) through group attacks similar to those of *V. mandarinia* (23, 33, 35) means that this giant vespine’s behavior and ecology merit further study beyond the fundamentals that we have explored here.

## Data availability statement

The original contributions presented in the study are included in the article/**Supplementary Material**. Further inquiries can be directed to the corresponding author.

## Ethics statement

Ethics approvals were not required for this study. We had verbal authorization for collections of nest contents from local residents who, according to Vietnamese law, had been granted land use rights on the properties where they were located. Giant hornets are not an endangered species, so no permits were required once permissions were granted.

## Author contributions

HM and GO conceived of the research. HM, GO, and LN acquired funding and conducted the field work in Vietnam. LN coordinated the collection of hornet nests and specimens. HM, LN, and GO made nest measurements; all authors contributed to measurements of hornet bodies. AP and MB conducted the geometric morphometric analyses of wings; HM conducted analyses related to genotyping and all other statistical analyses. HM wrote the first draft, with sections drafted by GO (nest measures methods and results) and AP and MB (geometric morphometric methods and results). All authors contributed to the article and approved the submitted version.
