# Supplementary material for: Biology of the southern giant hornet, Vespa soror: nest architecture, morphological differences among castes, and the genetic structure of colonies
Source: Front Insect Sci. 2023 Jul 6;3:1136297. doi: 10.3389/finsc.2023.1136297 (PMC10926378; doi:10.3389/finsc.2023.1136297)
Supplement: Supplementary file 2 [file Presentation_1.pdf]

Supplementary Material

Biology of the southern giant hornet, *Vespa soror*: nest architecture, morphological differences among castes, and the genetic structure of colonies

Heather R. Mattila*, Lien T.P. Nguyen, Adrien Perrard, Maggie Bain, and Gard W. Otis

*** Correspondence:** Heather R. Mattila: hmattila@wellesley.edu

1 Supplementary Data

Supplementary Data 1. All data produced in the analysis of the *V. soror* nests and hornet specimens are included in this Excel data file (for figures and tables in both the main text and Supplementary Materials). Additionally, we provide the SAS code (version 9.3) that was used for statistical analyses and the allele calls for the 8 microsatellites that were examined for workers from N1–N3 and the reproductives from N2.

2 Supplementary Video

Supplementary Video 1. A video of *V. soror* larvae scraping cell walls, a food-begging behavior known from other *Vespa* species. The video was taken 24 hours after adult hornets were removed from the combs of N1.

3 Supplementary Figures and Tables

3.1 Supplementary Figures

Supplementary Figure 1. Collection of N1 and N2 by vespiculturist Xuat Van Pham. Each spring and summer, Mr. Pham transfers small hornet colonies that he finds to his farm, where colonies continue their development undisturbed. He then sells the colonies in October and November to traders who supply hornet brood to restaurants in major cities, where it is consumed as a culinary delicacy. In 2013, he maintained approximately ten *V. soror* colonies, which he had transferred to brick-lined cavities that he created in embankments near his home. (A) To collect nests for us, he wore three layers of clothing, three pairs of rubber gloves, thick rubber boots, and a metal-screen "veil". He prepared a section of PVC tube to which he attached a large cage made of wire screening. To collect live hornets before excavating a nest, he slowly approached its entrance, into which he inserted the open end of the PVC tube, then he quickly sealed off gaps between the tube and the nest entrance with rags. As hornets exited their nest,

most of them flew up the tube towards the light and were collected in the screen cage. Once most of the adult wasps had been captured, that PVC tube was plugged with a rag that was then secured with tape. (B) After guiding hornets that remained in the nest into a second piece of PVC tube with a smaller wire cage affixed to the end, Mr. Pham used gloved hands to remove the nest from the ground. (C) One of the project's field assistants, Dai Dac Nguyen, holds the larger mesh cage full of live hornets at the conclusion of the field collection for N1. (D) Aerial *Vespa affinis* nests that Mr. Pham cultivated next to his family home.

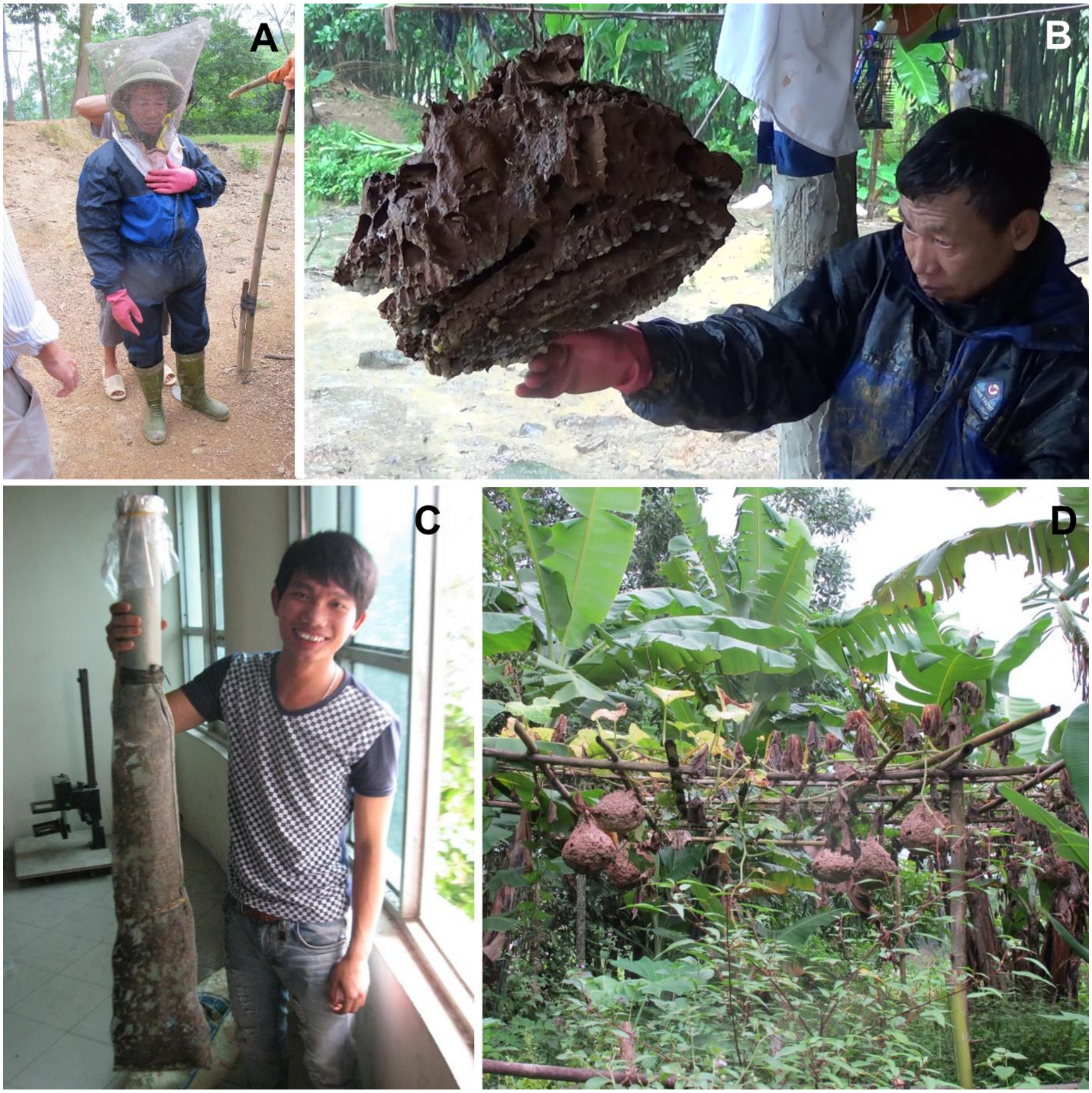

Supplementary Figure 2. Measurements taken from individual *V. soror* specimens. (A) Landmarks at intersections of wing veins used that were used for geometric morphometric analyses of female castes, adapted from Perrard et al. (44). Forewing length was measured as the distance from the base of the wing at the subcostal vein intersection to its apex (the dashed white line between the two points marked by X). For this study, we used methods similar to Perrard and Loope (45) to extract geometric morphometric data, for which forewings had their thick wing bases removed to avoid error produced by possible warping of the shape of the wing. However, our forewings were not originally prepared this way, so we tested whether the thick bases contributed to warping in images. After the initial pictures were taken and run through tpsDig2 software (with bases fully intact), a sample size of 10 workers was taken from each nest (n = 20 wings). Wing bases were removed, the wings were reimaged, and tpsDig2 software was used to digitize the 19 2D landmarks. Using the same R package 'geomorph' that was used in the original analysis, we conducted a Procrustes ANOVA with permutation to compare shape variation between the initial images with wing bases intact and the new pictures with the wing base removed (n = 10 images per group). No significant difference was found in wing shape between the two methods (P = 0.97). A further test of average shape variation of each group is equivalent to the square root of the sum of the variance of the 19 landmark coordinates for the wings. We found that wings with bases intact were less variable within their group (0.011) than wings with bases removed (0.017), and that removing the base of the wings did not reduce shape variation or make it a more accurate method of forewing preparation. (B) Dorsal view of a *V. soror* worker showing how the non-wing body measures were made on all *V. soror* individuals, including: head width (hw), thorax width (tw), metasomal tergite 1–3 widths (t1, t2, t3), and body length in dead specimens from the head to the apical margin of the second metasomal tergite (bl).

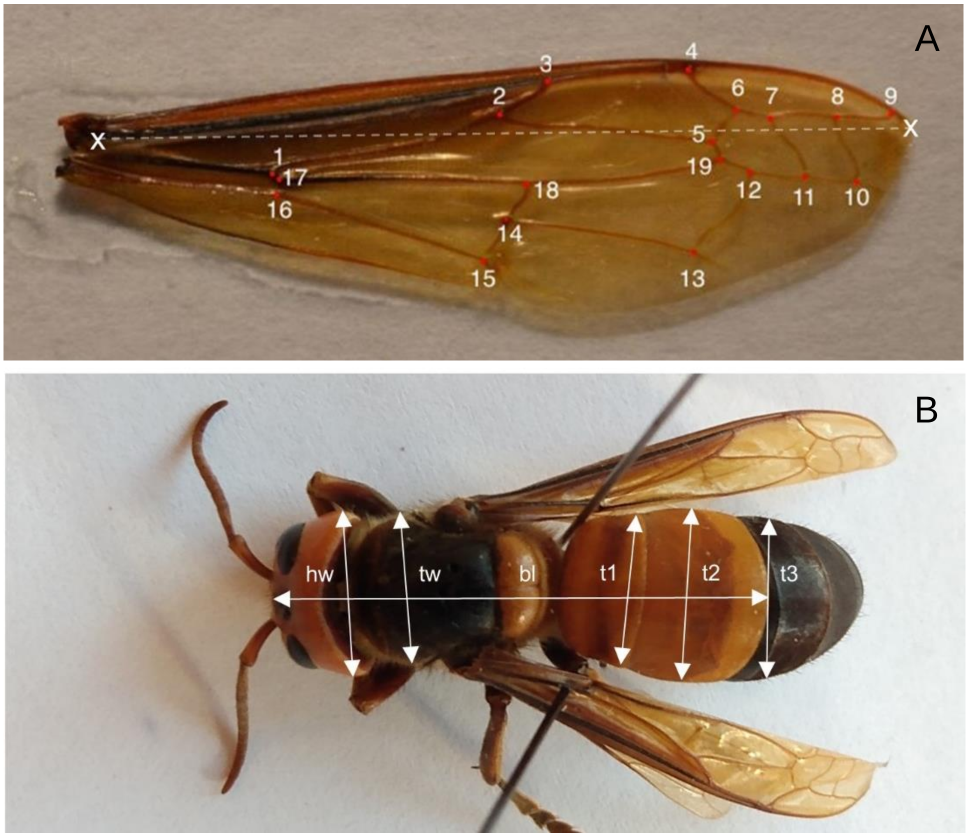

Supplementary Figure 3. There was a significant association between the area of a comb and the number of completed petioles that were constructed to attach it to the comb above (n = 8 combs across N1 and N2, Spearman' rank correlation: $r_s = 0.81$, P = 0.015). Petioles connecting comb 1 to the nest envelope were excluded for both nests; they could not be measured because they were destroyed when the envelopes were removed.

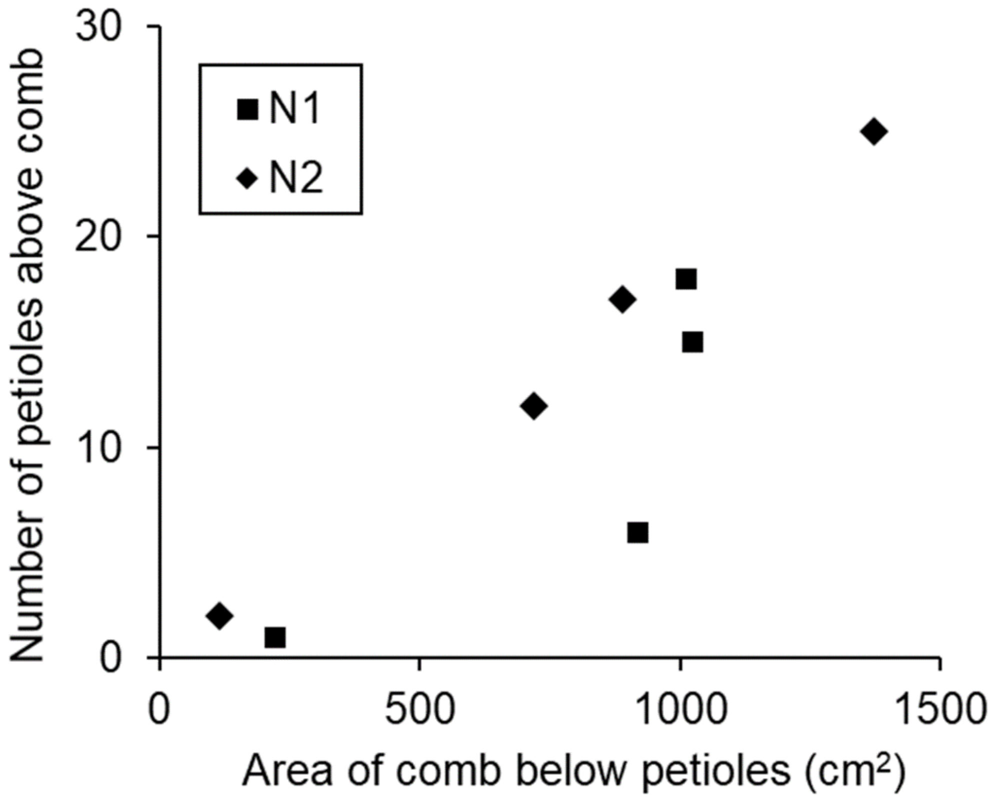

Supplementary Figure 4. Score plot for the first and second principal components from a PCA of wing vein landmarks measured from *V. soror* females from N2 (n = 85 workers and 44 gynes).

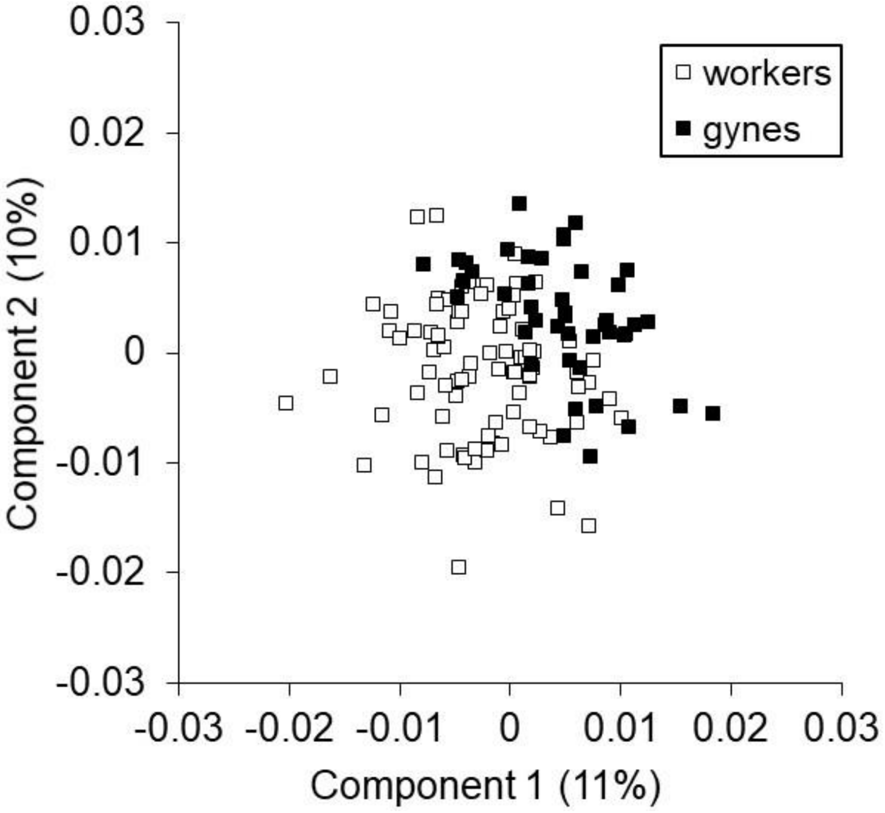

Supplementary Figure 5. Probability of not sampling a worker-produced male as a function of the percentage of all males that may be hypothetically produced by workers, according to Foster et al. (61).For N2, four loci had “informative alleles” (the workers’ father had an allele different than those of the queen). This information generates a probability of correctly assigning males as sons of the queen (N_a). For a sample of 30 males from N2, this probability translated to the likelihood that 28 out of 30 males could be correctly assigned as offspring of the queen (and 2 males being incorrectly assigned as sons of the queen, hence a non-detection error rate of 6.3%). These values inform the non-sampling error for males, which is $(1\text{-x})^{Na}$, where x is the proportion of the males that could be produced by workers. The figure shows that if workers produce a low percentage of males, then there is a high probability that a worker-derived male would be missed by our sample of 30 males from N2. Conversely, if workers produce a high proportion of males in N2, there is a low probability that a worker-produced male would have been missed by our sample.

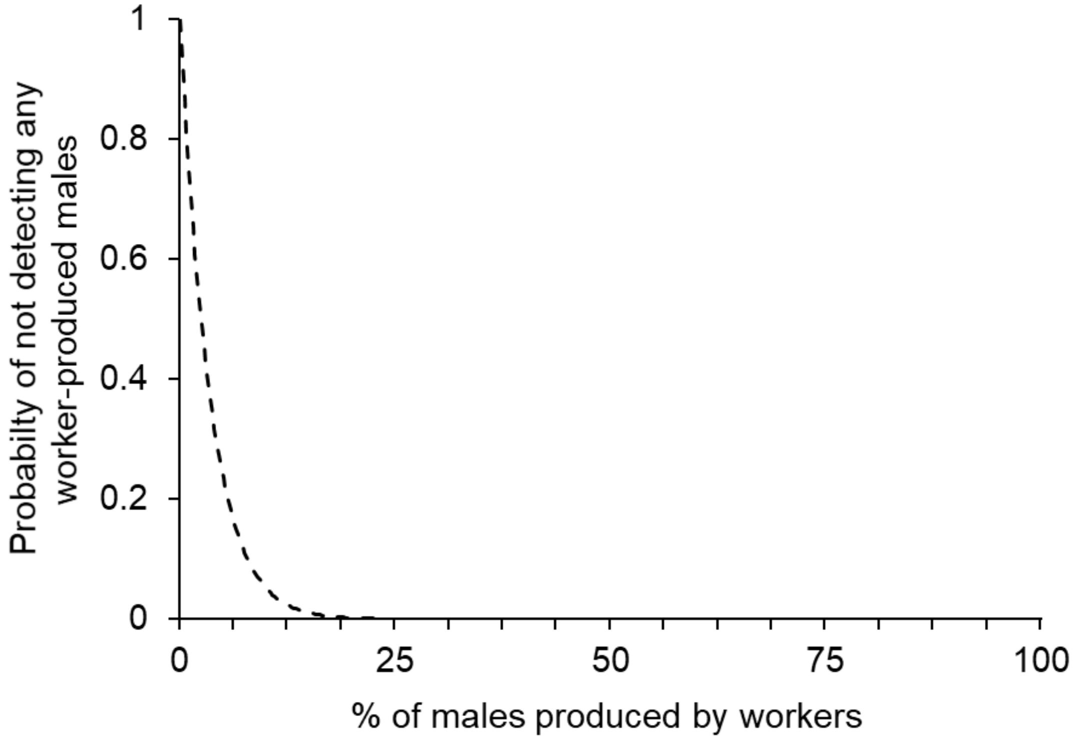

3.2 Supplementary Tables

Supplementary Table 1. Samples sizes and one-way ANOVA outcomes for the seven body measures that were made of *V. soror* specimens from N1–N3. Workers were collected from all nests; males were collected from N2 only. As many measures as possible were taken from each female (as specimen condition allowed). Only body and forewing length were measured from males. Body length measures were from the head to the apical margin of the second metasomal tergite. Asterisks indicate significant ANOVA outcomes. All statistical tests were highly significant, with gynes larger than workers in all instances (see Figures 5 and 6).

Measure	Sample size					ANOVA outcome		
	Workers N1	Workers N2	Workers N3	Gynes N2	Males N2	F-value	d.f.	P-value
Head width	100	86	122	45	n/a	77.7	3, 349	< 0.0001 *
Thorax width	100	86	122	45	n/a	104.1	3, 349	< 0.0001 *
Tergite 1 width	100	83	118	44	n/a	110.4	3, 341	< 0.0001 *
Tergite 2 width	100	83	118	44	n/a	117.5	3, 341	< 0.0001 *
Tergite 3 with	100	83	118	44	n/a	121.6	3, 341	< 0.0001 *
Forewing length	100	85	121	41	82	74.6	4, 424	< 0.0001 *
Body length	100	81	118	39	31	106.8	4, 364	< 0.0001 *

Supplementary Table 2. Dimensions of the petioles that attached horizontal combs in two *Vespa soror* nests (N1 and N2). In both nests, comb 1 is the uppermost and oldest comb and comb 5 is the lowest and newest comb. Petiole position is given with reference to the two combs they join. The diameter of each petiole was measured at the top and bottom where it attached to adjacent combs; diameter in each location was measured first at its widest point and then perpendicular to the first measurement. These two values were averaged to determine mean top and bottom diameters of each petiole; petiole height was measured as the distance between these attachment points. Means for all dimensions were calculated across petioles in each layer (standard deviations are in parentheses). Three petioles in N1 were partially constructed but failed to connect to a comb above; in these cases, only the bottom diameter was calculated for that petiole and smaller sample sizes are indicated for those layers. The bulky petioles connecting comb 1 to the nest envelope were damaged by the removal of the latter, so petiole dimensions for the top layer were not determined.

Petiole position	N1				N2			
	# of petioles	Diameter (top, mm)	Diameter (bottom, mm)	Height (mm)	# of petioles	Diameter (top, mm)	Diameter (bottom, mm)	Height (mm)
comb 1 to comb 2	16	8.5 (4.0) n = 15	11.3 (5.1)	11.0 (4.8)	25	9.3 (4.5)	9.8 (4.5)	14.0 (4.9)
comb 2 to comb 3	18	7.8 (3.2)	11.3 (8.1)	13.6 (4.7)	17	5.7 (3.5)	7.3 (3.7)	13.3 (4.8)
comb 3 to comb 4	8	6.8 (3.7) n = 6	11.7 (2.8)	13.7 (3.5)	12	5.8 (1.9)	5.7 (1.8)	13.8 (3.3)
comb 4 to comb 5	1	5.5	4.5	19.9	2	6.1 (0.5)	8.4 (1.5)	7.8 (1.9)
